# Bones

**Published:** 1870-04

**Authors:** 


### THE BISTOURY.

VOL. VI. ELMIRA, N. Y. APRIL, 1870. No. 1.

### Communications.

### BONES.

BY ISHMAEL.

ONE is not apt to become very well acquainted with his bones, unless he breaks one of them, or cuts through the flesh and lays one bare, and then, the acquaintance is not especially agreeable. Nevertheless, they are very necessary and useful in our anatomical construction. Without them, it would be a difficult matter to hold ourselves together, we would be so much like a wet rag or an oyster out of his shell. We would waddle around, if we moved at all, like animated currant jelly. Our bones, and especially our back-bones, hold us together, give a consistency and strength to our forms and assist us in locomotion. Bones are hard substances, as witness the knuckles of a man's hand when they come in contact with another's face, but the material that enters most largely into their construction is a liquid, this being that portion of the blood which upon exposure to the air, the quickest thickens. To this material, is added in proper quantities, a substance commonly called lime, not that our bones are at all like the mortar in which the bricks of our houses are laid or the plastering put upon our walls, although the lime operates in the same capacity in all three, that of hardening and solidifying them. People generally suppose that bones are white, probably gathering such a notion from specimens of these affairs that frequently strew the back-yards of many of our houses, but all bones have not this color. A species of fish have green bones and some varieties of the common fowl have black ones. Human bones, however, even of the negro, his color being only skin deep, are all white. The bones of the human body grow, not only from infancy to maturity, but during one's whole life. Hard as they are, they are continually changing, the blood furnishing the foundation for them and the digestive organs running in their supply of prepared lime. If the system is out of order and refuses to furnish the requisite supply of either material, it gives rise among children, to a disease which is called the rickets, in grown persons a softness of the bones. This is not, though it may seem to be, the matter of the favorite circus performer who is called the india rubber man. *He* has no bones at all, to speak of! Though we know the material of which bones are composed, that they are very near like common chalk, yet given the materials themselves, one would find it very difficult to manufacture them, if his supply should run out. Nevertheless, men are known to have made false legs!

Every individual, who is properly stocked for the customary business of life, is provided by Nature with one hundred and sixty-five different bones. Each bone to its own work. To an indifferent observer, it would seem that the number was large enough for every purpose, yet there have been instances where the bone making process has gone on so fast that excresences, bone-like in character, have appeared plentifully on the outside of the body. One man especially, had bones sticking out all over him and was called from this circumstance, the porcupine-man. It is proper to observe that this is not the case with antiquated spinsters or fashionable matrons who *will* persist in wearing low-necked dresses to exhibit their bones, these bones being only the usual supply allowed to women. If any one doubts the facts concerning bones herein set forth, let him examine himself and discover their falsehood or truthfulness, which is easy enough done, a consciousness of their existence being denied to no one, save perhaps the "fat boy" or the "mammoth lady." Human bones, however, are not very interesting subjects for the general reader; they are suggestive of death, the grave-yard and skeletons. The bones of animals are pleasanter subjects for contemplation, especially after one has plucked the meat from them, as is the case with the second joint of a turkey, the side bone of a duck, or a nice leg of mutton. There is where bones come as nicely into play as do those of the end man in a travelling minstrel troupe.

Bones are not always inside the body or covered with flesh. They are outside in the case of some animals, for the teeth, horns, shells, coral, ivory and other substances are but bone, either softer or harder, as the case or want may be, or as there is a greater or less amount of lime in their composition. And there are many curious circumstances in connection with them that are noticable. For instance, the antlers of the stag and elk which are real bones, and some of which are as much as eight feet long, and measure fourteen feet from tip to tip, are cast away annually, new ones growing out in twelve weeks! Some traveler in our western countries found large mounds of these antlers, piled up to an exceeding great heighth, and the wonder would naturally arise as to how the topmost one was put there by the animal. Considering the size of these horns and the material of which they are composed, it is safe to say that the stomachs of these creatures are the liveliest kind of lime-kilns in existence. In much the same manner, do the lobster and crab yearly cast off their shell overcoats aud assume new ones. They are like the man with only one shirt who had to go to bed while it was being washed. They retire to some lonely spot, where in their naked and defenceless state, they are less liable to be attacked by those who love lobster and crab; they throw away their old hard shells, and for a time become soft shells. I am not aware that any of these animals were ever caught at such periods. They should be, for the cooking of them would be a much less troublesome job. Soon, however, they go to work fashioning their new garments and in about a fortnight crawl forth again in new clothes.

One other fact in relation to these curious animals should be noted; they possess a power not enjoyed by any other species of being, that of reproducing an entire limb, if perchance one should be lost by accident or disease. How it is done, naturalists have not yet determined.

Belonging to bones are two substances very highly valued in nearly all countries, the one worn in some places as an amulet or charm, the other regarded with the greatest favor by the fair sex as being an object that lends an attraction to the most beautiful. The one is the charming and valuable bezoar stone, the other the pearl. Both nothing but bones, and the last worse than that, a diseased bone!

Of other sorts of bones, like those of birds, that are hollow like quills and made on purpose for flying, like those of the cuttle-fish, broader than those of any other aquatic animal, yet exceedingly light because exquisitely porous and cellular; like those of the beautiful paper nautilus, so delicate that they seem as feathers; like those of certain kinds of insects formed like a washboard, and upon which with their wings they make those noises that resemble cries from the mouth, of these and others, simple allusions to them are all that is necessary. Their uses and beauties are too well-known to need elaboration.

Bones too, being what might be called the frame-work of anatomy, and having set them up, I shall proceed shortly to build over and cover them in. In the language of BROUSSAIS, *Bon Soir*!

